# Na_v_igating the intricacies of cellular machinery

**DOI:** 10.1016/j.jbc.2021.100832

**Published:** 2021-05-26

**Authors:** Sara Nathan, Sandra B. Gabelli

**Affiliations:** 1 Department of Biophysics and Biophysical Chemistry, Johns Hopkins University School of Medicine, Baltimore, Maryland, USA; 2 Division of Cardiology, Department of Medicine, Johns Hopkins University School of Medicine, Baltimore, Maryland, USA; 3 Department of Oncology, Johns Hopkins University School of Medicine, Baltimore, Maryland, USA

**Keywords:** Na_v_, SCN, calmodulin, CaM, CALM1, calcium, FGF, FHF, ion channel, electrophysiology, CaM, calmodulin, CaMBD, CaM-binding domain, CTD, C-terminal domain, FGFs, fibroblast growth factors, LTP, long-term inactivation particle, Na_V_s, voltage-gated sodium channels

## Abstract

Voltage-gated sodium channels (Na_V_s) underlie the initiation of action potentials in various excitable cell types and are regulated by channel-interacting proteins, including the cellular calcium sensor calmodulin and fibroblast growth factor homologous factors. Both of these are known to bind the Na_V_ cytosolic C-terminal domain and modulate the channel’s electrophysiology, but it was unknown whether they had any allosteric interactions with each other. A recent rigorous study provides insights into the molecular interactions of these ion channels and their partners that crucially take the cellular landscape into consideration.

The critical role of voltage-gated sodium channels (Na_V_s) in cell excitation makes the intricacies of channel modulation of acute consequence, as evidenced by diseases including epilepsy, cardiac arrhythmias, and chronic pain among others that result from Na_V_ dysregulation (1). Na_V_s cycle rapidly between closed, open, and inactivated states, with channel-interacting proteins fine-tuning the transition between these functional states. Binding of fibroblast growth factor homologous factors (FGFs 11-14) has been associated with an increased rate of voltage-dependent fast inactivation as well as long-term inactivation, parameters that determine how quickly the inward sodium current is quelled and how long the channel is inactivated before it is able to open again, respectively (2). In parallel, CaM binds two Ca^2+^ on each of its two lobes and has been shown to interact with a highly conserved ‘IQ’ motif in the Na_V_ C-terminal domain (CTD) in both its calcium ion–free (apo-) calmodulin (CaM) and (Ca^2+^)_4_–CaM states. The functional consequence of the interaction between CaM and the Na_V_ CTD is dynamic and dependent on both the Na_V_ isoform and calcium status of CaM, with calcium-dependent channel inactivation being the best characterized (in skeletal muscle isoform Na_V_1.4 in particular; (3, 4, 5)). Although CaM and FGFs are known to interact with adjacent regions of the Na_v_ CTD, it was unknown whether CaM and FGFs interacted with each other as well, or whether they independently interfaced with the channel.

Although an indirect interaction between Na_V_-associated FGF and CaM has been suggested for Na_V_1.4, direct interaction and potential stoichiometry have not been explored until the Shea laboratory's extraordinary attention to detail brought the molecular mechanism into focus (6, 7, 8). The group has a strong history of bringing molecular studies to relevant physiological questions, including notably their description of the cooperativity of Ca^2+^ binding by CaM, of import to CaM’s extensive substrates. Originally described in the context of autophosphorylated CaMKII, this cooperative Ca^2+^ binding that underlies CaM’s change in conformation and leads to distinct substrate recognition is highlighted again by the Na_V_ complex (9). Mahling *et al.* have now demonstrated that the Na_V_ complex has multiple CaM-binding sites with differing calcium-saturating preferences, accentuating the nuanced and extremely dynamic nature of the interactions (6).

Here, Shea and colleagues identify two novel FGF sites that bind CaM. They term the first of these sites ‘long-term inactivation particle’ (LTP), as it has been shown to underlie the factors’ inactivation function (2, 10) and term the second ‘CaM-binding domain’ (CaMBD) (Fig. 1). They then thermodynamically and structurally characterized the calcium-dependent interaction with CaM at both sites before observing the interaction in complex with the Na_V_ CTD. The investigators performed a series of mutational studies to thoroughly assay CaM binding to the LTP and CaMBDs. They observed these interactions using fluorescence spectroscopy titrations and solution NMR and determined that both lobes of (Ca^2+^)_4_–CaM directly bind both FGF sites, whereas apo-CaM does not. They found (Ca^2+^)_4_–CaM binding to the aptly named CaMBD to range from about 5- to 44-fold higher than binding to the LTP, with variation among the FGF isoforms; the affinity of (Ca^2+^)_4_–CaM for the FGF CaMBD ranged from FGF12A with a *K*_*d*_ of 107 nM to FGF13A with a *K*_*d*_ of 13 nM (6).

**Figure 1:**
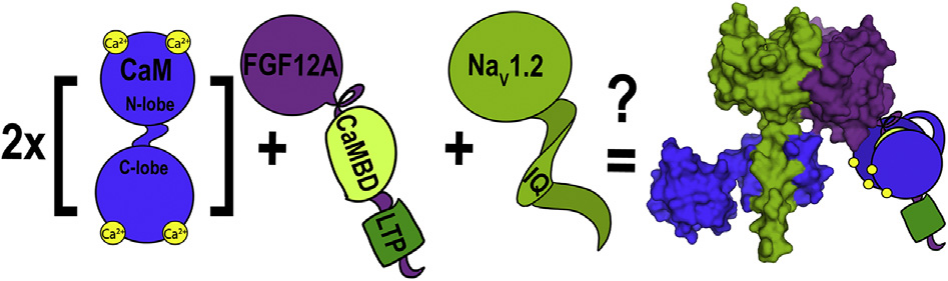
**The Shea group’s investigations revealed two novel CaM (*blue*) interaction domains on FGFs (*purple*; domains labeled CaMBD and LTP) with the CaMBD showing high affinity for calcium-saturated CaM.** It is known that the Na_V_ (*green*) IQ motif binds strongly to calcium-free (apo-)CaM. Together, these data suggest that upon CaM binding of calcium, the change in conformation results in CaM’s dissociation from the Na_V_ channel to reassociate with the proximal FGF. Alternatively, a second CaM molecule may be present in the complex (6). CaM, calmodulin; CaMBD, CaM-binding domain; FGFs, fibroblast growth factors; LTP, long-term inactivation particle; Na_V_s, voltage-gated sodium channels.

Although they found that the LTP and CaMBD sites could both bind CaM, reverse-phase HPLC of the (Ca^2+^)_4_–CaM complex with the larger N-terminal domain containing both binding sites suggested that the complex had a 1:1 CaM:FGF stoichiometry. This model was confirmed by solution NMR experiments in which (Ca^2+^)_4_–CaM binding to the entire N-terminal domain mirrored the shift observed from (Ca^2+^)_4_–CaM binding to the CaMBD peptide alone. With this information on the complex, the authors took the extra step of titrating free Ca^2+^ and observing the effect on binding by measuring intrinsic fluorescence to determine that the affinity of both CaM lobes for Ca^2+^ increased when CaM was in complex with the FGF12A CaMBD. Returning the study to the setting of the ternary complex, both CaM lobes again increased their Ca^2+^ binding affinity when FGF12A was added to the complex of CaM + Na_V_1.2CTD (a neural Na_V_ isoform). Given the two strong binding sites for CaM in this ternary complex (the FGF CaMBD and the Na_V_CTD ‘IQ’ motif), the authors repeated these free Ca^2+^ titrations with the [CaM]:[FGF12A]:[Na_V_1.2CTD] complex in a stoichiometric ratio of 2:1:1. The data obtained from a CaM mutant with only the C-lobe Ca^2+^-binding sites available resulted in a biphasic binding curve, suggesting that the CaM C-lobe was binding at two distinct sites. These data were contrasted with the curve from the CaM N-lobe mutant, which formed a monophasic binding curve; this is contiguous with numerous reports of the CaM C-lobe anchoring the molecule’s binding to the Na_V_ CTD (6).

Mahling *et al.* take these comprehensive thermodynamic and structural data together with previous studies describing CaM binding to Na_V_ (with greatest affinity in its apo-CaM form) to suggest a possible mechanism in which an action potential results in elevated cytosolic Ca^2+^ concentrations, causing apo-CaM bound to Na_V_ to bind Ca^2+^, change conformation to disassociate, and then reassociate with the proximal FGF. They note it is also a definite possibility that the FGF recruits a second CaM molecule to the complex (6). The true stoichiometry likely varies depending on the cellular context. In the cell, these distinctions of Ca^2+^ dynamic signaling are highly consequential when considering the framework of an action potential, initiated by Na_V_, that results in a rapid transient cytosolic increase in Ca^2+^ concentrations to trigger muscle contraction, the release of synaptic vesicles, or other cellular functions. This work should inspire the field to reconsider the stoichiometry of the macromolecular complexes central to neuron firing and muscle excitation–contraction. The Shea laboratory's investigations into cooperative ligand interactions and the conformational changes induced by CaM binding provide these critical links that illuminate the intricacies of cellular machinery.

## Conflict of interest

The authors declare that they have no conflicts of interest with the contents of this article.
